# Mitomycin-C Needle Bleb Revision in Congenital Glaucoma

**DOI:** 10.4103/0974-9233.71598

**Published:** 2010

**Authors:** Thanaa Helmy Mohamed Elsayed, Tamer Mohamed El-Raggal

**Affiliations:** Department of Ophthalmology, Faculty of Medicine, Ain Shams University, Cairo, Egypt

**Keywords:** Congenital Glaucoma, Mitomycin-C, Needling

## Abstract

**Purpose and Settings:**

This study was designed to evaluate the efficacy and safety of mitomycin-C (MMC) augmented needling procedure in the management of failed bleb after trabeculectomy in congenital glaucoma. This study was carried out at Ain Shams University Hospital.

**Patients and Methods:**

A retrospective study was carried on 30 eyes of 25 patients with congenital glaucoma with bleb failure after trabeculectomy. The mean age of the subjects was 7.3 ± 3.4 years (range, 1–12 years). Under general anesthesia, needling procedure was performed with adjunctive use of a mixture of 0.1 mL of MMC (0.04 mg/mL) and 0.2 mL of lidocaine 1% injected subconjunctivally. Needling was performed with a 30-gauge needle to dissect the areas of subconjunctival fibrosis and re-establish aqueous outflow.

**Results:**

Follow-up ranged from 6 to 20 months (mean, 9.23 ± 5.25 months). One needling revision was performed in 22 eyes (73.3%) and eight eyes (26.7%) received two needle revisions. The mean intraocular pressure (IOP) decreased from 26.9 ± 2.85 mmHg (range, 21–34 mmHg) before surgery to 15.63 ± 3.15 mmHg (range, 10–24 mmHg) at last follow-up. Complications included significant subconjunctival hemorrhage in six eyes, intraoperative bleb leak in two eyes, choroidal detachment in one eye, and minimal hyphema in one eye.

**Conclusion:**

MMC needle bleb revision appears to be an effective method to revive failed filtration surgery after trabeculectomy in patients with congenital glaucoma. This technique is effective in reducing IOP with preservation of the remaining conjunctiva for further surgery.

## INTRODUCTION

Congenital glaucoma results from a developmental defect of anterior chamber angle tissue derived from neural crest cells leading to aqueous outflow obstruction. The primary intervention to control the intraocular pressure (IOP) in congenital glaucoma is surgical.^1^ Surgery aims at eliminating the resistance to aqueous outflow created by structural abnormalities in the anterior chamber angle. This can be achieved with either an internal drainage procedure such as goniotomy or external drainage procedure such as trabeculotomy or trabeculectomy.^2^

Trabeculectomy, when done primarily has been shown to have good results in congenital glaucoma.^3^ It has the advantage of being a procedure that is commonly performed by most ophthalmologists. However, primary trabeculectomy is not entirely straightforward in congenital glaucoma as the eye is large and the limbal anatomy is distorted.^4^ Even years after successful trabeculectomy for adult glaucoma, there is a high likelihood that a significant proportion of patients will require further surgery within their lifetimes.^5^

The introduction of mitomycin-C (MMC) has significantly enhanced the success of trabeculectomy in glaucomatous adults at high risk of surgical failure. However, the response of very young children to MMC augmented trabeculectomy is extremely variable, with some patients scarring rapidly despite antifibrotic therapy, whereas others develop hypotony with large avascular filtering bleb.^6^

Needling revision is an option in cases of failed primary trabeculectomy for congenital glaucoma when the IOP is high. One or more needling procedures can be performed if there is an open sclerostomy site and attempts are made to re-open the filtering site by cutting through the fibrotic subconjunctival and intrascleral tissue. An additional injection of antimetabolites may prevent formation of new scar tissue. This combined technique of antimetabolite injection and needling has been used with variable success.^6^

The aim of this study is to evaluate the efficacy and safety of MMC augmented needling procedure in the management of failed bleb (either flat or encapsulated) after trabeculectomy in congenital glaucoma.

## MATERIALS AND METHODS

### Patients

A retrospective study was performed on 30 eyes of 25 patients (14 males and 11 females) with congenital glaucoma with bleb failure after a single trabeculectomy. Institutional review board approval was obtained prior to data collection.

Inclusion criteria included a history of trabeculectomy and the presence of a filtering bleb with open sclerostomy confirmed by gonioscopy. We performed needling for eyes that had previously undergone trabeculectomy with or with antimetabolites. The present authors had no clinical bearing on the primary procedure. The following descriptive parameters were collected: age, gender, type of glaucoma, time elapsed since trabeculectomy, IOP measurement that was repeated on the day of needling, number of antiglaucoma drugs, and bleb morphology prior to surgery.

### Needling procedure with MMC

Bleb needling was performed under general anesthesia in all cases with sterile technique for the entire procedure. Sterilization and lid draping were performed in a regular fashion for intraocular surgery. Gonioscopy was carried out to ensure an open trabecular window followed by IOP measurement with a handheld Perkins Applanation Tonometer (Haag Streit AG, Koeniz, Switzerland). A mixture of 0.1 mL of MMC (0.04 mg/mL) mixed with 0.2 mL of nonpreserved 1% lidocaine was withdrawn into 1 cc syringe. A 30-gauge needle was mounted on the syringe and bent bevel up at the hub achieving an angle of 30°–45°.

The needle was inserted into the subconjunctival space 5–8 mm lateral to the scleral flap. The mixture was injected elevating the conjunctiva, then the needle was advanced until the tip of the needle was inside the filtering bleb. A sweeping motion was used to create an incision in the fibrous tissue overlying the flap. Gentle pressure with the needle bevel was exerted to elevate the scleral flap if there was transscleral fibrosis. In the case of an encapsulated bleb, several openings of the fibrous capsule were created with the needle. The location of the needle tip was moved in a manner that avoided cutting blood vessels or perforating the globe or the conjunctiva.

Successful removal of outflow obstruction resulted in an immediate increase in the area of the bleb or decrease in the height of encapsulated bleb with escape of aqueous into the surrounding subconjunctival space. The needle was then withdrawn, and the conjunctival entry point was examined for leakage using the Seidel test. If leakage was present, the area was first dried and then sealed with handheld cautery or one 8-0 vicryl stitch. One to two drops of topical broad-spectrum antibiotic (Okacin, Novartis Ophthalmics, Basel, Switzerland) were applied at the end of the procedure. Postoperatively, a combination of tobramycin and dexamethasone 0.1% (Tobradex, Alcon, Fort Worth, TX, USA) was prescribed four times daily for 2 weeks. The parents were instructed to avoid direct pressure on the globe and valsalva maneuvers for at least 2 weeks (the child was obliged to remain in bed for the majority of the time and avoid straining, e.g., crying and coughing).

IOP was measured at first week, second week, then every 2 weeks for 2 months, then every month after surgery. IOP was measured under good sedation using chloral hydrate in the operating room. Fundus examination was performed to assess the optic nerve and to detect choroidal detachment.

The procedure was considered successful if the IOP less than or equal to 18 mmHg without the use of antiglaucoma medications. A qualified success was defined if IOP was less than or equal to 18 mmHg with the aid of 1 or 2 antiglaucoma medications. A failure was defined as inability to satisfy the above criteria. Reneedling using the same technique was performed after 2–4 months if the initial needling was not successful in creating adequate filtration and IOP control.

The statistical analysis was carried out using SPSS version 10 for Windows (SPSS Inc., Chicago, IL). Student’s *t*-test for paired data was used to compare preoperative and postoperative results.

## RESULTS

The mean age of the patients was 7.3 ± 3.4 years (range, 1–12 years). The sample included 14 males and 11 females. The right-to-left eye ratio was 17:13. Out of the 30 eyes included, twenty-eight eyes were diagnosed with primary glaucoma, 1 eye with Sturge Weber syndrome, and 1 eye as neurofibromatosis. Sixteen eyes (53.3%) had a flat bleb, and 14 eyes (46.7%) had encapsulated bleb. Failure after the primary trabeculectomy occurred by 5.72 ± 1.39 months (range, 1.5–12 months). The mean follow-up was 9.23 ± 5.25 months (range, 6–20 months) after needling.

There was statistically significant decrease in the IOP after needling with MMC (*P* < 0.01). The mean preoperative IOP was 26.9 ± 2.85 mmHg (range, 21–34 mmHg), whereas the mean postoperative IOP was 15.63 ± 3.15 mmHg (range, 10–24 mmHg) at last follow-up. There was a marked decrease of IOP after needling with a mean IOP of 16.5 ± 3.21 mmHg, 1 week postoperatively, but the mean IOP slightly increased by the third month (18.0 ± 2.87 mmHg) and then decreased again after re-needling in some cases and introduction of medical treatment to other cases (15.63 ± 3.15 mmHg).

**Figure 1:**
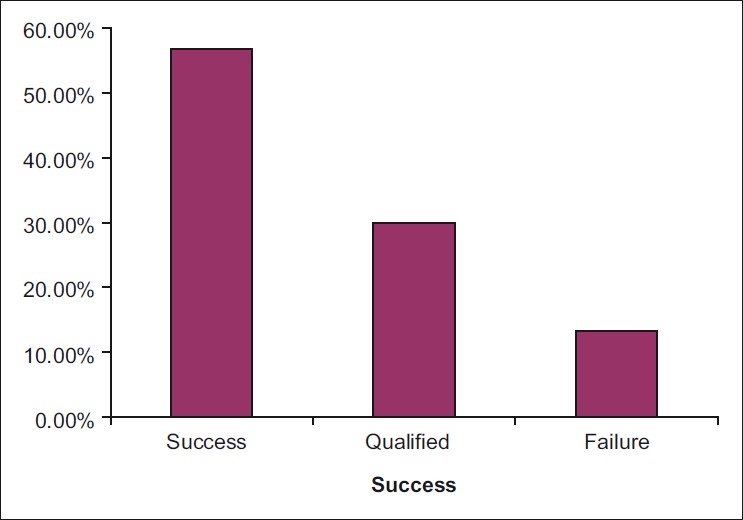
Final results in needle bleb revision

Of the 30 eyes included, 22 eyes (73.3%) underwent one needle revision and eight eyes (26.7%) underwent two needle revisions. For the eight eyes that underwent a second needle revision, four eyes (50%) succeeded and the remaining four eyes failed. Needle revision was successful in 17 cases (56.7%), qualified success occurred in nine cases (30%), and failure occurred in four cases (13.3%) [Figure 1]. Higher success rate was obtained in eyes with a mean preoperative IOP 24.9 ± 1.7 mmHg.

Topical antiglaucoma drops succeeded in controlling IOP for a mean of 9.23 months in subjects requiring medical treatment after needling.

There was a reduction in the number of medications required by the subjects after needling [Table 1]. Seventeen eyes (56.7%) did not need medication after needling, nine eyes (30%) needed only one medication and four eyes needed two medications (13.3%) to achieve control of IOP. The mean number of postoperative medications was 0.64 ± 0.76 medications.

**Table 1:** Comparison between the preoperative and postoperative number of medications

Number of medications	Mean	Paired *t*	*P* value
Preoperative	1.39 ± 0.62	7.02	<0.01
Postoperative	0.64 ± 0.76		

Intraoperative complications included significant subconjunctival hemorrhage in six eyes (20%), and minor hemorrhage (self-limiting small subconjuctival hemorrhage without conjunctival ballooning or blood entry under the scleral flap) in 20 eyes (66.67%). Intraoperative bleb leak was observed in two eyes (6.67%), one of which required suturing and the other required cauterization.

Postoperative complications included hypotony with choroidal detachment in one eye (3.3%) with neurofibromatosis that recovered spontaneously within 2 weeks with normalization of IOP. Minimal hyphema was observed in one eye of another patient (3.3%) that resolved within 3 days. Other sight threatening complications such as corneal toxicity, suprachoroidal hemorrhage, malignant glaucoma, and endophthalmitis were not encountered in this series.

## DISCUSSION

Failure of filtration surgery can occur early or may be delayed for months or even years. Early failure may result from surgical complications, technical errors or simply from a vigorous healing response especially in young patients. The use of antimetabolites has reduced the frequency of bleb failure due to scarring but has resulted in thinner blebs that are more susceptible to infection and leakage.

Management of bleb failure depends on the cause and the interval since the primary surgery. In some cases, it may be prudent to attempt modification of the original procedure in the hope of a more favorable response. Since 1941, many authors have described the bleb needling procedure for adult glaucoma using various methods yet the fundamental principle remains the same. The success rates reported for needling revision procedures on adults have been variable. Studies show that 5-Fluorouracil (5-FU) needling revision is an effective and safe method of restoring patency to previously fibrosed passage of aqueous flow within failed filtration blebs. However, more than one revision is often required and success might be limited in some patients despite repeated revisions.^7^ The use of MMC as an adjunct to needle revision may be helpful especially when high-risk factors for failure exist.^7^

To our knowledge, this is the first study of a series of failed congenital glaucoma surgery that were managed with MMC augmented needling surgery. Hence, comparison to similar studies is not possible. However, data on the adult glaucoma population have been published. Previously published series of needling of failed bleb after trabeculectomy in adult glaucoma report success rates ranging between 64.2% and 94% with varying criteria of success.^7^–^9^ Using different antimetabolites, Hodge *et al*.^8^ reported success in 94.1% of subjects who received 5-FU while Mardelli *et al*.^9^ reported success in 75.8% of eyes that underwent slit lamp needling augmented with MMC. In comparison to 5-FU, MMC is a more potent inhibitor of fibroblast proliferation and can be used intraoperatively, making it an attractive alternative for cases of previously failed surgery.^10^ We selected to use MMC because, compared to 5-FU, it is less toxic to the corneal epithelium, more effective and does not require follow-up injections.

Our success rate of 86.7% in congenital glaucoma cases is similar to or exceeds previous experience on adults with glaucoma requiring needling. Shin *et al*.^11^ reported success in 80% of cases requiring needling with 5-FU one or more times after previously failed filtering bleb. Costa *et al*.^12^ reported success in 64.2% of cases after one needling procedure. They defined a complete success as IOP less than 20 mmHg after one treatment, whereas we defined success as IOP less than or equal to 18 mmHg irrespective of the number of treatments. This difference in the definition of success likely accounts of the different success rate in our study and in their study.^12^

In our study, we performed needling twice in 26.7% of eyes. The repeated needling with MMC increased the success rate to 86.7%. Additionally, there was a significant reduction in the overall number of antiglaucoma medications required after MMC needling revision. Similarly, Perucho-Martinez *et al*.^13^ reported needling twice in 17.39% of the eyes with a success rate of 25% after the second procedure. In our study, a successful outcome resulted in 50% of eyes that underwent two needle revisions. On the basis of this outcome, needling can be repeated more than twice if required.

Although a technically simple procedure, major complications have been reported with needling. These include hypotony, suprachoroidal hemorrhage, malignant glaucoma, and endophthalmitis.^9^ Complications in this study were similar to those previously reported on an adult population. For example, Greenfield *et al*.^14^ reported 3.17% eyes with hypotony. Hypotony occurred in one eye (3.3%) in our cohort of cases, which recovered within 2 weeks with normalization of IOP. Mardelli *et al*.^9^ reported 3.2% eyes with hyphema immediately after needling resolved within 1 week. In our study, minimal hyphema was observed in one eye (3.3%) that resolved within 3 days. Greenfield *et al*^14^ who reported wound leak in 6.3% of eyes after needling. Mardelli *et al*.^9^ reported bleb leak in 8.1% of cases. Similarly, we found 6.6% of eyes had wound leakage from the site of needle entry all of which were successfully managed.

Endophthalmitis has been reported in children who have undergone trabeculectomy with MMC.^15^ In adults, the incidence of endophthalmitis following antimetabolite use ranges from 2% to 9%.^15^ Sidoti *et al*. described higher incidences of infectious complications (10% blebitis and 7% endophthalmitis), probably due to the higher MMC concentration (0.5 mg/mL) utilized in their study.^16^ They observed a relatively long interval between surgery and the development of infectious complications with the majority of endophthalmitis cases occurring 24 months after surgery.^16^

Our experience of needling in congenital glaucoma cases presented here indicates that the results are not substantially different from previously published studies on adult glaucoma. No specific precautions or postoperative complications occur that were different from the adult population. Although long-term topical treatment is not recommended in children, topical therapy did succeed in controlling the IOP for 9.23 months after needling. This allowed the surgeons to consider further intervention in cases of long-term noncompliance or drug failure.

The limitations of our study is the relatively small sample of only 30 eyes and the short mean follow-up period of only 9.23 months. This study was not designed to evaluate late complications after needling. The risk of long-term complications with this technique and the requirement for further surgical intervention warrant investigation. Another limitation is that visual acuity was not reported in this pediatric cohort, where drop in vision remains an important concern. Our criteria of success were based on IOP control whereas vision preservation is an important variable with all ophthalmic surgery. Future studies need to incorporate visual acuity testing after needling in congenital glaucoma.

In conclusion, needle bleb revision with MMC appears to be a relatively effective, safe, and simple procedure for congenital glaucoma. Since needling is performed at the same site of the trabeculectomy, it preserves the conjunctiva for a possible subsequent trabeculectomy in this young population. We recommend needling as an alternative in the management of failed filtration blebs, in eyes with congenital glaucoma associated with inadequate IOP control. Further studies with larger samples size and longer follow-up are recommended.
